# Variation of Detailed Protein Composition of Cow Milk Predicted from a Large Database of Mid-Infrared Spectra

**DOI:** 10.3390/ani9040176

**Published:** 2019-04-18

**Authors:** Marco Franzoi, Giovanni Niero, Giulio Visentin, Mauro Penasa, Martino Cassandro, Massimo De Marchi

**Affiliations:** 1 Department of Agronomy, Food, Natural Resources, Animals and Environment, University of Padova, Viale dell’Università 16, 35020 Legnaro (PD), Italy; marco.franzoi89@gmail.com (M.F.); mauro.penasa@unipd.it (M.P.); martino.cassandro@unipd.it (M.C.); massimo.demarchi@unipd.it (M.D.M.); 2 Associazione Nazionale Allevatori della Razza Frisona e Jersey Italiana, Via Bergamo 292, 26100 Cremona, Italy; giuliovisentin@anafi.it

**Keywords:** parity, days in milk, lactation, casein, lactoglobulin

## Abstract

**Simple Summary:**

Milk proteins are one of the most valuable milk components. The objective of the present study was to assess sources of variation of detailed protein composition predicted from infrared spectra in milk of dairy and dual-purpose cattle breeds. Results showed that protein fractions were primarily influenced by days in milk, and the relative proportion of each fraction through lactation was not constant. Protein fractions correlated with crude protein, total casein, fat and milk urea nitrogen. In perspective, mid-infrared predictions of milk fractions could be useful for the dairy sector to improve nutritional and technological properties of milk.

**Abstract:**

This study aimed to investigate factors affecting protein fractions, namely α-casein (α-CN), β-casein (β-CN), κ-casein (κ-CN), β-lactoglobulin (β-LG) and α-lactalbumin (α-LA) predicted from milk infrared spectra in milk of dairy and dual-purpose cattle breeds. The dataset comprised 735,328 observations from 49,049 cows in 1782 herds. Results highlighted significant differences of protein fractions in milk of the studied breeds. Significant variations of protein fractions were found also through parities and lactation, with the latter thoroughly influencing protein fractions percentage. Interesting correlations (*r*) were estimated between β-CN, κ-CN and β-LG, expressed as percentage of crude protein, and milk urea nitrogen (*r* = 0.31, −0.20 and −0.26, respectively) and between α-LA and fat percentage (*r* = 0.41). The present study paves the way for future studies on the associations between protein fractions and milk technological properties, and for the estimation of genetic parameters of predicted protein composition.

## 1. Introduction

Milk proteins are one of the most valuable components among milk constituents. This is mainly due to the wide array of nutritional, nutraceutical and technological properties they are endowed with. First, milk and dairy products are major sources of proteins in the human diet, both in terms of recommended daily intake and biological value [1]. Second, milk and whey proteins, and peptides derived from their metabolic hydrolysis, have nutraceutical properties, such as antibacterial, antiviral, antifungal and antioxidant activity [2]. Adequate milk protein intake, together with calcium and vitamin D, results in decreased bone fracture and osteoporosis risk [3]. Third, caseins are primarily involved in the cheese-making process, since they are the only milk constituents reacting to rennet and are mainly responsible for milk coagulation properties and yield, retaining also other milk components and water in the caseinate complex [4]. Casein fractions influence milk coagulation properties; in particular, κ-casein (κ-CN) and α-casein (α-CN) proportions have positive effects on curd firming time and curd firmness [5]. At the same time, whey proteins have been reported to influence curd properties, for example, α-lactalbumin (α-LA) has been demonstrated to improve the rate of firming and curd firmness, contrary to β-lactoglobulin (β-LG) [6].

For all these reasons, milk protein content is included in the quality-based payment systems of many dairy companies [7] as well as in the selection indexes of different breeds and countries [8]. Accordingly, the possibility of characterizing not only total protein or total casein content but also specific protein fractions at population level could be of great interest in order to genetically improve the milk aptitude to coagulate, considering the influence that milk proteins have on milk coagulation properties and cheese yield [5]. Quantification of total milk proteins and caseins is based on the Kjeldahl method, whereas qualitative and quantitative analyses of detailed milk protein composition are based on High Performance Liquid Chromatography (HPLC) [9,10,11]. Such techniques, commonly recognised as reference or gold standard methods, are not adequate for the acquisition of phenotypic information at population level due to their high demand in terms of costs, time and trained personnel [12]. For these reasons, large-scale collection of protein fractions is still partially hampered, thus preventing their inclusion in breeding programmes and in quality-based payment systems. Mid-infrared spectroscopy (MIRS) has been recognized as a reliable, fast and cost-effective tool for the prediction of milk phenotypes, including total protein and casein content [13]. Moreover, an advantage of MIRS is the possibility to retroactively apply calibration models and thus study the temporal variation of novel traits when spectra are properly stored and standardized [14]. Recently, the feasibility of characterizing detailed milk protein composition using mid-infrared prediction models has been investigated [11], and population-level studies have been conducted [15,16].

To our knowledge, there is a paucity of information on the fine protein composition of cow milk predicted from mid-infrared spectra at population level. Therefore, the objectives of the present study were to (i) assess sources of variation of detailed milk protein composition predicted by MIRS in a large database of dairy and dual-purpose cattle breeds, and (ii) estimate the correlations between the milk content of protein fractions and other milk traits.

## 2. Materials and Methods 

### 2.1. Data Collection

Data and spectra information of 2,119,143 milk analyses of fat, crude protein (CP) and casein percentage, and milk urea nitrogen (MUN, mg/dL) collected between January 2011 and December 2017 were provided by the South Tyrolean Dairy Association (Bolzano, Italy). Milk yield (kg/day) and somatic cell count (SCC, cells/µL) were also available. Information on herds and cows were provided by the Breeders Association of Bolzano Province (Bolzano, Italy). Milk samples were from 128,328 Holstein-Friesian (HF), Brown Swiss (BS), Simmental (SI), Alpine Grey (AG) and Pinzgauer (PI) cows farmed in 4453 single-breed herds. The average size of herds under milk recording in this mountainous area is small and animals are fed forage or hay and concentrates. Between 15% and 20% of the farms move their cows to highland pastures in late spring or early summer, and during the highland sojourn animals have access to grazing.

Immediately after collection, 50 mL of milk samples were added with 200 μL of preservative (Bronysolv; ANA.LI.TIK Austria, Vienna, Austria) and processed in the laboratory of the South Tyrolean Dairy Association according to the guidelines of the International Committee for Animal Recording for milk quality analyses. Fat, CP and casein percentages, and MUN content were determined using MilkoScan FT6000 or MilkoScan FT7 (FOSS Electric A/S, Hillerød, Denmark). To offset changes in instrumental response and ensure the comparability of spectra between MilkoScan FT6000 and MilkoScan FT7, the two instruments were routinely calibrated using a standard sample, according to the manufacturer instructions [17]. Principal component analysis on spectra did not show significant differences between the two instruments. Somatic cell count was determined using a Cell Fossomatic (FOSS Electric A/S, Hillerød, Denmark) and transformed to somatic cell score (SCS) with the following formula: SCS = log_2_ (SCC/100) + 3. Spectral data from 5000 to 900 cm^−1^ were used to develop MIRS models to predict detailed milk protein composition.

### 2.2. MIRS Calibration Models

Detailed milk protein composition was predicted using equations developed by Niero et al. (2016) [11]. Briefly, 114 samples from the same area of the present study were collected and analysed for α-CN, β-casein (β-CN), κ-CN, β-LG and α-LA contents through HPLC (Agilent 1260 Series; Agilent Technologies, Santa Clara, CA, USA). Samples preparation and protein fractions separation were carried out following the procedure of Maurmayr et al. (2013) [18]. Spectral regions corresponding to water noise absorption (1700 to 1600 cm^−1^ and 3660 to 3040 cm^−1^) were discarded. Calibrations were developed using SAS software ver. 9.4 (SAS Institute Inc., Cary, NC, USA). Partial least squares regression analysis coupled with uninformative variable elimination procedure was performed following the approach developed by Gottardo et al. (2015) [19]. Ratio performance deviation in leave-one-out cross-validation (root mean square error in cross-validation) was 2.86 (1.05 mg/mL) for α-CN, 1.60 (0.53 mg/mL) for β-CN, 2.03 (0.88 mg/mL) for κ-CN, 1.34 (1.10 mg/mL) for β-LG and 1.30 (0.10 mg/mL) for α-LA [11]. For the purpose of the present study, protein fractions were expressed in absolute concentration (mg/mL) and as a percentage of CP.

### 2.3. Data Editing and Statistical Analyses

In the present study, days in milk (DIM) between 5 and 305 days, and parity between 1 and 15 were considered. Lactations with less than three test day records were discarded from the dataset. Observations from cows that changed herd during the investigated period were removed. The final dataset consisted of 735,328 records from 49,049 cows and 1782 single-breed herds, collected between January 2011 and December 2017 during the official monthly test day recording. Records were from two dairy (HF, *n* = 6271 cows; BS, *n* = 15,556 cows) and three dual-purpose cattle breeds (SI, *n* = 16,836 cows; AG, *n* = 9202 cows; PI, *n* = 1184 cows). Spectra outliers were identified by calculating the Mahalanobis distance between the data point (spectrum) and the centroid of the spectra cluster. Predicted milk protein fractions were set to missing if outside the range of the reference data used for calibrations. For all studied traits, values deviating more than 3 standard deviations from the corresponding trait mean were set to missing.

Sources of variation of detailed milk protein composition and traditional milk traits were investigated using the HPMIXED procedure of SAS software ver. 9.4 (SAS Institute Inc., Cary, NC, USA), according to the following linear model:

y*_ijklmno_* = µ + B*_i_* + M*_j_* + Y*_k_* + S*_l_* + P*_m_* + (B × M)*_ij_* + (B × S)*_il_* + (B × P)*_im_* + (S × P)*_lm_* + H*_n_*(B*_i_*) + C*_o_*(B*_i_*) + e*_ijklmno_*

where y*_ijklmno_* is the analysed trait; µ is the overall intercept of the model; B*_i_* is the fixed effect of the *i*th breed (*i* = HF, BS, AG, SI, PI); M*_j_* is the fixed effect of the *j*th month of sampling (*j* = 1 to 12); Y*_k_* is the fixed effect of the *k*th year of sampling (*k* = 2011 to 2017); S*_l_* is the fixed effect of the *l*th DIM class of the cow (*l* = 1 to 30; 10-day classes); P*_m_* is the fixed effect of the *m*th parity of the cow (*m* = 1 to 5, with class 5 including cows of parity ≥ 5); (B × M)*_ij_* is the fixed interaction effect between breed and month of sampling; (B × S)*_il_* is the fixed interaction effect between breed and DIM class; (B × P)*_im_* is the fixed interaction effect between breed and parity; (S × P)*_lm_* is the fixed interaction effect between DIM class and parity; H*_n_*(B*_i_*) is the random effect of the *n*th herd nested within the *i*th breed ~N(0,σ^2^_H(B)_); C*_o_*(B*_i_*) is the random effect of the *o*th cow nested within the *i*th breed ~N(0,σ^2^_C(B)_); and e*_ijklmno_* is the random residual ~N(0,σ^2^_e_). Because of the data structure (herd nested within breed), the significance of the breed effect was tested on herd within breed variance. A multiple comparison of means was performed for the main effect of breed, using Bonferroni’s test (*p* < 0.05). Finally, Pearson correlations between residuals of milk production traits and detailed protein composition were assessed using the CORR procedure of SAS.

## 3. Results and Discussion

In the present study, only data from single-breed herds were available for statistical investigation. No detailed information on diet and management of the cows was available; however, feeding strategies of the herds were based on requirements and production levels of their breeds, and thus the breed-estimated effect could also include a part of the farming conditions (herd) effect. For this reason, a nested approach has been used, similarly to previous papers [20,21].

### 3.1. Descriptive Statistics

Descriptive statistics and proportion of phenotypic variance accounted by cow and herd effects for milk yield, composition, SCS, MUN and detailed milk protein composition are reported in Table 1. Milk yield averaged 23.45 kg/day, and means of fat, CP, casein, SCS and MUN were 4.03%, 3.46%, 2.72%, 2.48 and 21.19 mg/dL, respectively. Averages of milk yield and composition traits observed in the present study were comparable with values reported by Penasa et al. (2014) [22], who studied milk coagulation properties of HF, BS and SI cows in multi-breed herds, and Visentin et al. (2018) [21], who assessed the phenotypic variation of major milk mineral content in HF, BS, AG and SI cows in single-breed herds.

Means for protein fractions were 14.30, 10.45, 7.30, 1.82 and 0.70 mg/mL of milk for α-CN, β-CN, κ-CN, β-LG and α-LA, respectively, and the corresponding means for protein fractions expressed as percentage of CP were 41.36%, 30.48%, 21.25%, 5.23% and 2.02%, respectively (Table 1). Quantifications of milk proteins obtained in the present study were consistent with detailed protein composition determined by HPLC in the milk of HF and Jersey breeds [23]. The amount of total κ-CN was slightly greater than that reported by other authors, and this was probably due to the high incidence of BS cows in the present study (32% of total animals) and to farming systems that favoured milk composition rather than milk yield [24].

The greatest proportion of phenotypic variance explained by herd effect was estimated for milk yield (35.09%) and MUN (20.27%), meaning that farm management and feeding system were important for these features. For all other traits, the cow was more important than the herd effect in explaining the phenotypic variation; in particular, values ranged from 25.58% (fat percentage) to 41.78% (casein percentage) for milk quality traits, and among milk protein fractions they were lowest for α-LA and greatest for β-LG, regardless of the unit of measurement (Table 1). Overall, the result for β-LG reflects the fact that protein and its fractions are only partially affected by variations in nutrition and management [25]. Considering that this protein fraction has been identified as one of the major milk allergens, strategies such as genetic selection might be of particular interest to decrease its content in milk and develop hypoallergenic milk and functional foods [26].

### 3.2. Breed Effect

To our knowledge, this is one of the first studies that has used historical spectra information to predict protein composition in different dairy and dual-purpose cattle breeds. Bonfatti et al. (2017) [9] studied milk protein composition using predicted protein phenotypes from a large spectra database of Italian SI cows. Even if some studies about milk protein composition have been published recently, all of them investigated the phenotypic and genetic variation of milk protein composition using HPLC on a limited number of samples. Moreover, concerning the two dual-purpose breeds (AG and PI), their detailed protein composition has been characterized for the first time in the present study.

Table 2 reports the least squares means (LSMs) of milk yield, composition, SCS, MUN and detailed protein fractions for HF, BS, SI, AG and PI breeds. Alpine Grey and HF had the lowest (17.10 kg/day) and the highest milk yield (28.43 kg/day), respectively. Regarding chemical composition, fat, CP and casein percentages were greater for BS cows than for other breeds, and SI cows had significantly lower SCS (2.45) than other breeds, with SCS from 2.62 (AG) to 2.85 (BS). Milk urea nitrogen ranged from 19.04 mg/dL (HF) to 21.74 mg/dL (AG). Overall, detailed milk protein composition varied significantly across breeds. In particular, BS cows showed the greatest amount of all casein fractions and the lowest amount of β-LG when expressed as mg/mL (*p* < 0.05), whereas HF exhibited the lowest amount of caseins, even if not significantly different from PI, and α-LA. The greatest β-LG content (mg/mL) was observed in the milk of SI cows (*p* < 0.05). 

Cipolat-Gotet et al. (2018) [27] determined detailed milk protein composition of 1264 Italian BS samples through reversed phase HPLC and results showed that protein fraction contents were similar to those reported in the current study, except for κ-CN, which will be discussed more in details later on, and β-LG. Differences in the latter were probably determined by the wider lactation range in the study of Cipolat-Gotet et al. (2018) [27] compared with the present work.

In order to investigate differences in the relative proportion of protein fractions, LSMs were estimated for proteins expressed as g/100 g of CP. As a result, α-CN differed slightly among breeds, with values between 41.12% (AG) and 41.75% (SI), whereas β-CN, κ-CN and α-LA were significantly greater in BS (31.81%, 21.99% and 2.10%, respectively) compared with other breeds. The lowest concentration of β-CN (29.28%) was estimated for SI, and the lowest concentration of κ-CN was obtained for HF (20.76%) and SI (20.81%). Finally, β-LG ranged from 4.34% (BS) to 5.91% (HF).

Relative proportions of α-CN and β-LG percentage in HF breed (41.64% and 5.91%, respectively) were lower compared with results of Schopen et al. (2009) [28] in first-parity Dutch HF cows, whereas β-CN was higher compared with the same study (30.44% and 27.17%, respectively). Such differences can be attributed to the different cow parities and lactation stages included in the sampling, to diversities in farming system and area, and to the lower relative amount of κ-CN observed in the study of Schopen et al. (2009) [28]. Those authors determined only non-glycosylated mono-phosphorylated κ-CN using capillary zone electrophoresis, and this can explain the lower κ-CN percentage compared with that obtained in our study. Such hypothesis is corroborated by κ-CN determined in the study of McDermott et al. (2017) [15], which is consistent with the κ-CN reported in the present study. Previous reports predicted protein fractions content of the SI cattle breed from infrared spectra. Compared with Bonfatti et al. (2017) [9], lower α-CN and β-LG and higher β-CN and κ-CN were found in the present study. Such differences could be attributed to the same factors already discussed for HF.

### 3.3. Effects of Parity, Lactation Stage and Season

**Figure 1:**
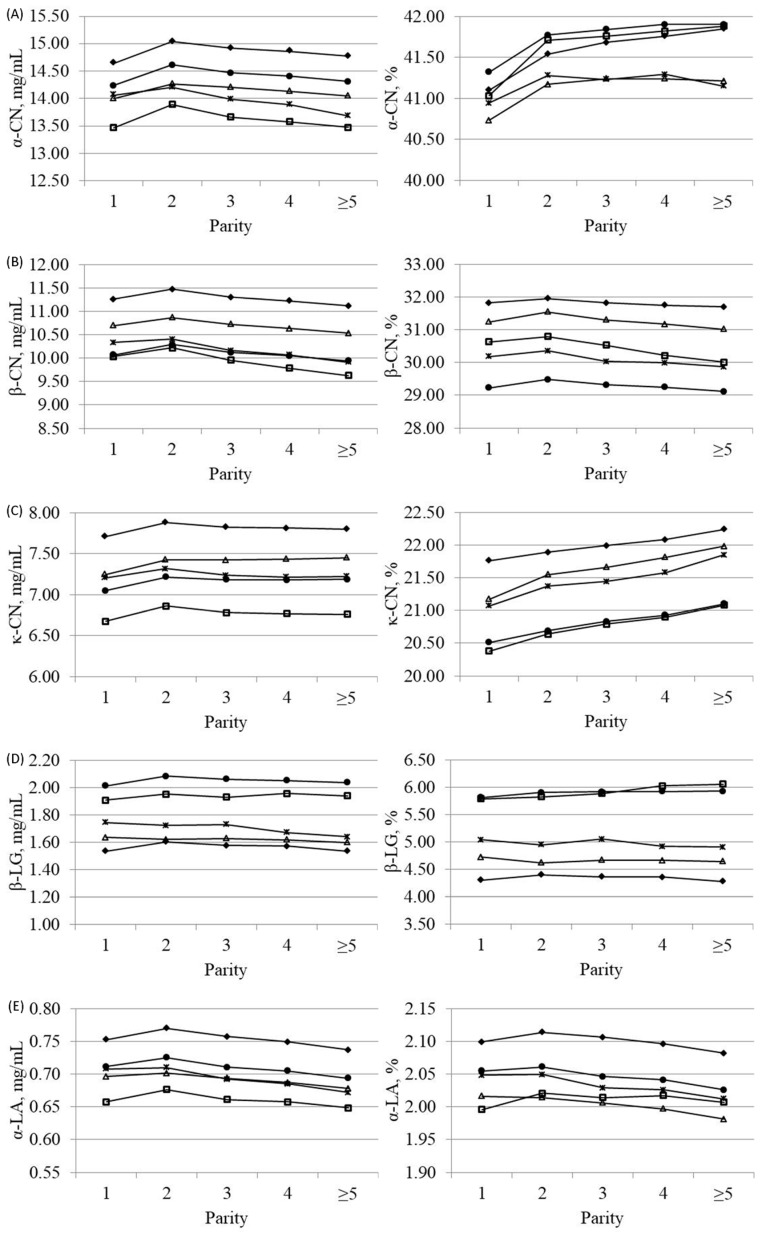
Least squares means of (**A**) α-casein (α-CN), (**B**) β-casein (β-CN), (**C**) κ-casein (κ-CN), (**D**) β-lactoglobulin (β-LG) and (**E**) α-lactalbumin (α-LA) across parity for Brown Swiss (-♦-), Holstein-Friesian (-□-), Alpine Grey (-△-), Simmental (-●-) and Pinzgauer (-×-) cows, expressed as mg/mL of milk (on the left side) or percentage of crude protein (on the right side).

Variations of protein fractions across different parities and breeds are depicted in Figure 1. All caseins and α-LA, expressed as mg/mL of milk, followed a trend similar to that of CP (Supplementary Figure S1), with the greatest amount in second-parity cows and a decreasing content in later parities. The same trend was not so clear for β-LG, which showed only slight variations across different parities. Switching to protein fractions expressed as percentage of CP, α-CN and κ-CN increased in milk of older compared with first- and second-parity cows, with a more obvious trend for specialized dairy breeds (HF and BS). Conversely, β-CN and α-LA decreased with parity order, and β-LG remained almost stable.

**Figure 2:**
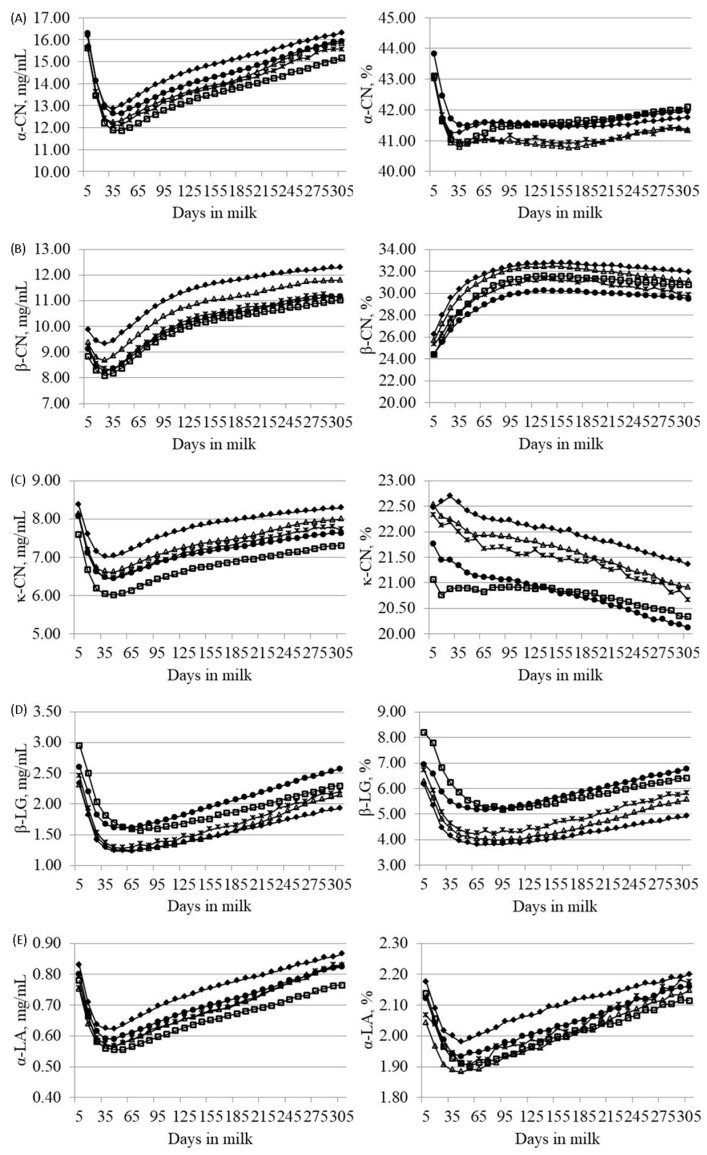
Least squares means of (**A**) α-casein (α-CN), (**B**) β-casein (β-CN), (**C**) κ-casein (κ-CN), (**D**) β-lactoglobulin (β-LG) and (**E**) α-lactalbumin (α-LA) across lactation for Brown Swiss (-♦-), Holstein-Friesian (-□-), Alpine Grey (-△-), Simmental (-●-) and Pinzgauer (-×-) cows, expressed as mg/mL of milk (on the left side) or percentage of crude protein (on the right side).

Figure 2 depicts the LSMs of predicted protein composition across lactation for HF, BS, AG, SI and PI breeds. Overall, the trend of milk protein composition measured as mg/mL across DIM mirrored that of CP (Supplementary Figure S2). Interestingly, protein fractions percentages showed important variations across DIM. In particular, α-CN decreased from 5 to 45 DIM and then slightly increased until 305 DIM, with different trends among breeds, and β-CN increased until 125 to 155 DIM and slightly decreased thereafter. A constant decrease of κ-CN was observed through the entire lactation, with a more gradual slope for HF. The variation of milk protein fractions across lactation may explain the trend of milk technological properties described in previous reports on the same breeds and study area [29,30]. Finally, β-LG and α-LA decreased until 75 DIM and increased during the remaining part of the lactation. Such trends for β-LG and α-LA resemble those recorded by Niero et al. (2016) [24] and Maurmayr et al. 2018 [31] who measured β-LG and α-LA using HPLC. Higher percentage of β-LG in early lactation could be associated with the biological function of this protein fraction in newborn calves, with particular regard to its ability to increase the absorption of small hydrophobic ligands such as retinol and fatty acids [32].

**Figure 3:**
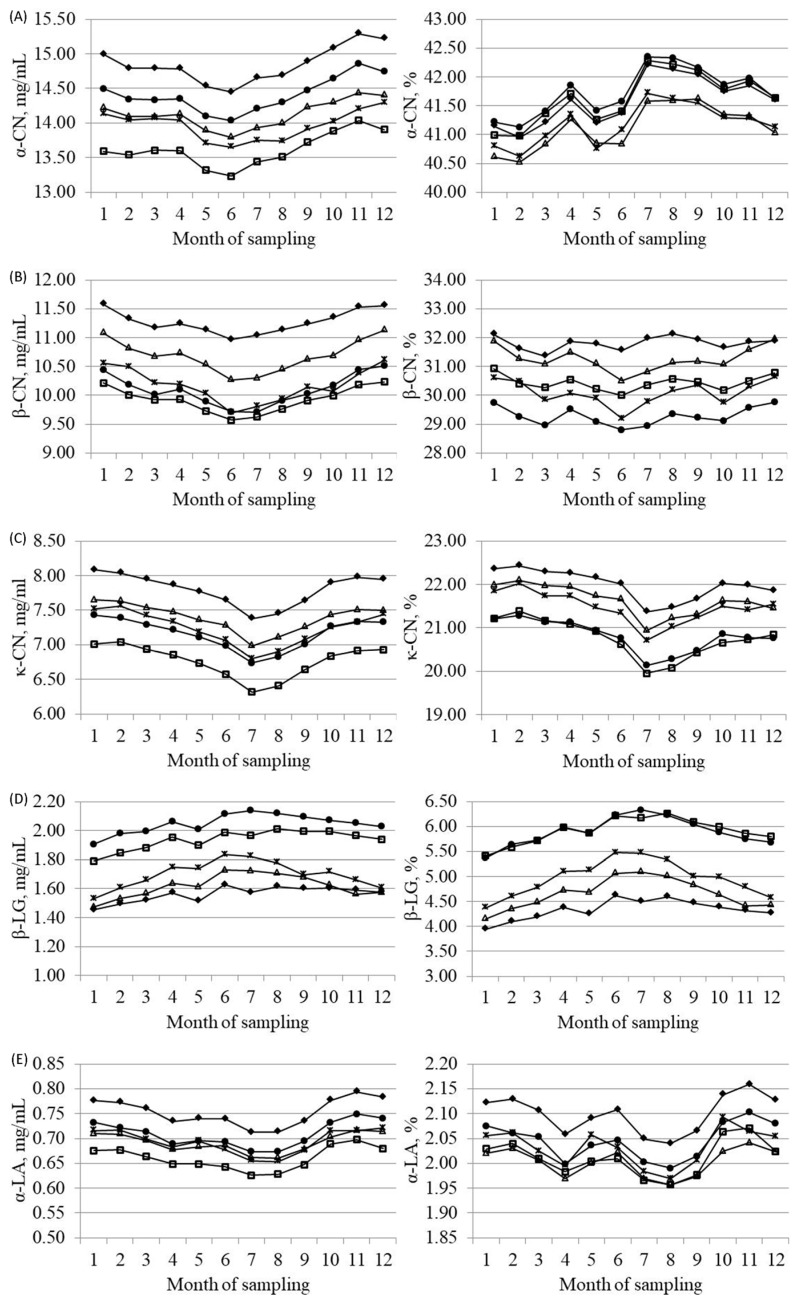
Least squares means of (**A**) α-casein (α-CN), (**B**) β-casein (β-CN), (**C**) κ-casein (κ-CN), (**D**) β-lactoglobulin (β-LG) and (**E**) α-lactalbumin (α-LA) across month of sampling for Brown Swiss (-♦-), Holstein-Friesian (-□-), Alpine Grey (-△-), Simmental (-●-) and Pinzgauer (-×-) cows, expressed as mg/mL of milk (on the left side) or percentage of crude protein (on the right side).

Regarding monthly variation of the amount of protein fractions (mg/mL of milk), caseins and α-LA followed the same trend as CP (Supplementary Figure S3), with a general decrease during the summer period and the minimum in June–July (Figure 3). Such trend was previously reported by Bernabucci et al. (2015) [33] and was correlated to heat stress affecting cows during summer. On the contrary, β-LG increased during the summer period, probably due to its immunomodulatory role. Percentage of α-CN showed two major peaks in April and July, whereas κ-CN (%) slightly decreased during summer, with a minimum in July, and β-CN (%) exhibited only small variations across months of sampling. Finally, β-LG (%) slightly increased between May and September, and α-LA (%) had an erratic trend, with the greatest percentage in November. Similar trends for protein fractions were reported by Bernabucci et al. (2015) [33]. Seasonal impacts on protein fractions could be the result of the pasture system applied in alpine areas during summer [34].

### 3.4. Correlations

All Pearson correlations (*r*) between the studied traits were different from zero (*p* < 0.001; Table 3). Correlations between protein fractions expressed as mg/mL ranged from −0.21 (β-LG and β-CN) to 0.56 (α-CN and β-CN, and β-CN and κ-CN). Moderate relationships were observed between α-CN and κ-CN (*r* = 0.52), and α-CN and α-LA (*r* = 0.42), and weak correlations were observed between β-LG and other protein fractions (*r* = −0.21 to 0.24). Regarding protein fractions expressed as percentage of CP, correlations ranged from −0.53 (β-CN and β-LG) to 0.37 (β-CN and κ-CN). In particular, β-LG was moderately negatively correlated with β-CN (*r* = −0.53) and κ-CN (*r* = −0.42), and κ-CN was moderately positively correlated with β-CN (*r* = 0.37).

Protein fractions expressed as mg/mL were moderately to strongly associated with CP and total casein (*r* = 0.32 to 0.89), and weakly negatively associated with milk yield (*r* = −0.16 to −0.06; Table 3), which is consistent with a dilution effect of milk components at higher milk yield [35]. Overall, milk protein fractions were also weakly associated with fat percentage, SCS and MUN (*r* = −0.24 to 0.26), except for a moderate correlation between α-LA and fat percentage (*r* = 0.41). Correlations of protein fractions, expressed on CP, with milk yield, composition, SCS and MUN were generally weak (*r* = −0.26 to 0.31), except for a moderate relationship between α-LA and fat percentage (*r* = 0.41; Table 3). Differences in the magnitude of correlations between MUN and milk protein fractions probably underline that each protein fraction has a different impact on nitrogen conversion efficiency [36].

## 4. Conclusions

The prediction of detailed milk protein composition from milk mid-infrared spectra provided the opportunity to characterize sources of variation and phenotypic correlations for such important economic traits. The present study focused on five cow breeds, two dairy (BS, HF) and three dual-purpose (SI, AG, and PI), in single-breed herds. As a result, breed and lactation stage largely affected milk protein composition. Among the studied breeds, milk of BS showed the greatest amount of caseins, in particular κ-CN, which is important due to its impact on cheese-making properties. Further studies will focus on the estimation of genetic parameters for protein fractions and on the effect of milk protein composition on technological traits at population level.

## Figures and Tables

**Table 1:** Mean, standard deviation (SD), range, coefficient of variation (CV) and percentage of phenotypic variance accounted by cow (σ^2^_c_) and herd (σ^2^_h_) for milk yield, milk composition, somatic cell score (SCS), milk urea nitrogen (MUN) and detailed protein composition of cow milk.

Traits	Mean	SD	Range	CV (%)	σ^2^_c_ (%)	σ^2^_h_ (%)
Milk yield (kg/day)	23.45	7.41	44.70	31.61	24.63	35.09
Milk composition (%)						
Fat	4.03	0.65	4.49	16.16	25.58	7.65
Crude protein	3.46	0.38	2.45	11.06	40.62	17.48
Casein	2.72	0.30	1.90	10.93	41.78	17.40
SCS	2.48	1.78	11.18	72.02	29.52	8.88
MUN (mg/dL)	21.19	7.17	43.60	33.83	14.70	20.27
Protein fractions (mg/mL)						
α-casein	14.30	1.78	10.88	12.43	35.67	18.12
β-casein	10.45	1.64	10.03	15.71	37.56	10.50
κ-casein	7.30	0.96	5.91	13.18	36.53	11.57
β-lactoglobulin	1.82	0.77	4.29	42.45	44.76	7.22
α-lactalbumin	0.70	0.15	0.22	21.43	22.67	6.98
Protein fractions (% of crude protein)						
α-casein	41.36	1.90	35.80	4.59	21.46	9.58
β-casein	30.48	3.69	47.81	12.12	36.15	9.63
κ-casein	21.25	2.12	34.34	9.97	28.30	13.18
β-lactoglobulin	5.23	2.13	18.64	40.82	45.63	6.83
α-lactalbumin	2.02	0.31	3.25	15.47	8.11	2.62

**Table 2:** Least squares means (SE in parentheses) of milk yield, milk composition, somatic cell score (SCS), milk urea nitrogen (MUN) and detailed protein composition of different cow breeds ^1^.

Traits	Brown Swiss	Holstein-Friesian	Simmental	Alpine Grey	Pinzgauer
Milk yield (kg/day)	23.33 (0.13) ^a^	28.43 (0.26) ^b^	23.19 (0.14) ^a^	17.10 (0.17) ^c^	19.99 (0.55) ^d^
Milk composition (%)					
Fat	4.20 (0.01) ^a^	4.02 (0.01) ^b^	4.05 (0.01) ^b^	3.84 (0.01) ^c^	4.00 (0.03) ^b^
Crude protein	3.58 (0.01) ^a^	3.27 (0.01) ^b^	3.45 (0.01) ^c^	3.44 (0.01) ^d^	3.40 (0.02) ^d^
Casein	2.81 (0.01) ^a^	2.56 (0.01) ^b^	2.71 (0.01) ^c^	2.70 (0.01) ^c^	2.67 (0.02) ^c^
SCS	2.85 (0.02) ^a^	2.73 (0.04) ^ab^	2.45 (0.02) ^c^	2.62 (0.03) ^b^	2.79 (0.09) ^ab^
MUN (mg/dL)	21.64 (0.13) ^a^	19.04 (0.26) ^b^	20.22 (0.14) ^c^	21.74 (0.16) ^a^	20.19 (0.54) ^abc^
Protein fractions (mg/mL)					
α-casein	14.85 (0.03) ^a^	13.62 (0.05) ^b^	14.41 (0.03) ^c^	14.13 (0.03) ^d^	13.96 (0.11) ^bd^
β-casein	11.27 (0.02) ^a^	9.93 (0.04) ^b^	10.09 (0.02) ^c^	10.69 (0.02) ^d^	10.18 (0.08) ^bc^
κ-casein	7.81 (0.01) ^a^	6.77 (0.02) ^b^	7.16 (0.01) ^c^	7.40 (0.01) ^d^	7.24 (0.05) ^cd^
β-lactoglobulin	1.56 (0.03) ^a^	1.94 (0.02) ^b^	2.05 (0.01) ^c^	1.62 (0.01) ^d^	1.70 (0.04) ^d^
α-lactalbumin	0.75 (0.01) ^a^	0.66 (0.01) ^b^	0.71 (0.01) ^c^	0.69 (0.01) ^d^	0.69 (0.01) ^cd^
Protein fractions (% of crude protein)					
α-casein	41.59 (0.02) ^a^	41.64 (0.04) ^ab^	41.75 (0.02) ^b^	41.12 (0.03) ^c^	41.18 (0.09) ^c^
β-casein	31.81 (0.04) ^a^	30.44 (0.09) ^b^	29.28 (0.05) ^c^	31.25 (0.06) ^d^	30.08 (0.18) ^b^
κ-casein	21.99 (0.03) ^a^	20.76 (0.05) ^b^	20.81 (0.03) ^b^	21.63 (0.03) ^c^	21.46 (0.11) ^c^
β-lactoglobulin	4.34 (0.02) ^a^	5.91 (0.05) ^b^	5.89 (0.02) ^b^	4.66 (0.03) ^c^	4.97 (0.10) ^c^
α-lactalbumin	2.10 (0.01) ^a^	2.01 (0.01) ^bc^	2.05 (0.01) ^d^	2.00 (0.01) ^b^	2.03 (0.01) ^cd^

^1^ Least squares means with different superscript letters within a row are significantly different (*p* < 0.05).

**Table 3:** Pearson correlations, calculated using linear model residuals, between milk yield (MY), milk composition, somatic cell score (SCS), milk urea nitrogen (MUN), and detailed protein composition of cow milk.

Trait ^1^	MY, kg/d	Fat, %	CP, %	Casein, %	SCS	MUN, mg/dL	α-CN, mg/mL	β-CN, mg/mL	κ-CN, mg/mL	β-LG, mg/mL	α-LA, mg/mL	α-CN, %	β-CN, %	κ-CN, %	β-LG, %
Fat, %	−0.03														
CP, %	−0.18	0.14													
Casein, %	−0.14	0.17	0.98												
SCS	−0.11	0.08	0.10	0.07											
MUN, mg/dL	0.02	0.04	0.02	−0.02	−0.02										
α-CN, mg/mL	−0.14	0.15	0.89	0.86	0.07	0.04									
β-CN, mg/mL	−0.06	−0.02	0.53	0.48	−0.01	0.26	0.56								
κ-CN, mg/mL	−0.06	0.20	0.58	0.55	0.09	−0.15	0.52	0.56							
β-LG, mg/mL	−0.16	−0.06	0.32	0.34	0.06	−0.24	0.24	−0.21	−0.11						
α-LA, mg/mL	−0.08	0.41	0.46	0.48	0.07	0.02	0.42	0.18	0.30	0.10					
α-CN, %	−0.02	0.11	0.23	0.20	−0.01	0.07	0.64	0.30	0.14	−0.04	0.13				
β-CN, %	0.04	−0.11	−0.05	−0.10	−0.07	0.31	0.05	0.81	0.27	−0.49	−0.11	0.20			
κ-CN, %	0.07	0.15	−0.12	−0.14	0.04	−0.20	−0.11	0.24	0.72	−0.41	−0.01	−0.04	0.37		
β-LG, %	−0.13	−0.09	0.12	0.15	0.04	−0.26	0.06	−0.35	−0.24	0.97	0.01	−0.09	−0.53	−0.42	
α-LA, %	−0.02	0.41	0.13	0.16	0.03	0.02	0.13	−0.01	0.11	−0.01	0.93	0.05	−0.10	0.03	−0.03

Abbreviations are as follows: CP, crude protein; α-CN, α-casein; β-CN, β-casein; κ-CN, κ-casein; β-LG, β-lactoglobulin; α-LA, α-lactalbumin. All correlations are different from zero (*p* < 0.001).

## Supplementary Materials

The following are available online at https://www.mdpi.com/2076-2615/9/4/176/s1, Figure S1: Least squares means of (a) crude protein (CP), (b) casein and (c) milk urea nitrogen (MUN) across parity for Brown Swiss (-♦-), Holstein-Friesian (-□-), Alpine Grey (-△-), Simmental (-●-) and Pinzgauer (-×-) cows; Figure S2: Least squares means of (a) crude protein (CP), (b) casein and (c) milk urea nitrogen (MUN) across lactation for Brown Swiss (-♦-), Holstein-Friesian (-□-), Alpine Grey (-△-), Simmental (-●-) and Pinzgauer (-×-) cows, Figure S3: Least squares means of (a) crude protein (CP), (b) casein and (c) milk urea nitrogen (MUN) across month of sampling for Brown Swiss (-♦-), Holstein-Friesian (-□-), Alpine Grey (-△-), Simmental (-●-) and Pinzgauer (-×-) cows.
