# Supplementary material for: Variation of Detailed Protein Composition of Cow Milk Predicted from a Large Database of Mid-Infrared Spectra
Source: Animals (Basel). 2019 Apr 18;9(4):176. doi: 10.3390/ani9040176 (PMC6523433; doi:10.3390/ani9040176)

animals

Supplementary files: Variation of Detailed Protein Composition of Cow Milk Predicted from a Large Database of Mid-Infrared Spectra

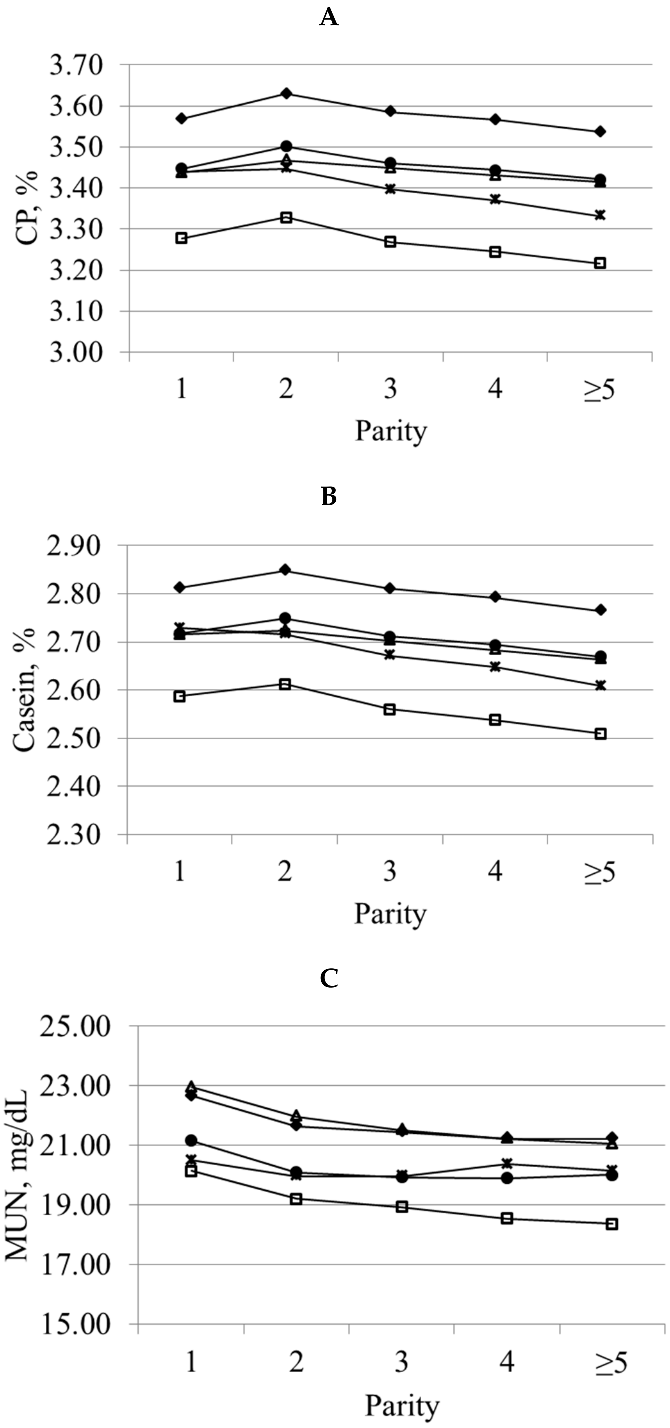

Figure S1. Least squares means of (**A**) crude protein (CP), (**B**) casein and (**C**) milk urea nitrogen (MUN) across parity for Brown Swiss (-♦-), Holstein-Friesian (-□-), Alpine Grey (-△-), Simmental (-●-) and Pinzgauer (-×-) cows.

Figure S2. Least squares means of (**A**) crude protein (CP), (**B**) casein and (**C**) milk urea nitrogen (MUN) across lactation for Brown Swiss (-♦-), Holstein-Friesian (-□-), Alpine Grey (-△-), Simmental (-●-) and Pinzgauer (-×-)

A

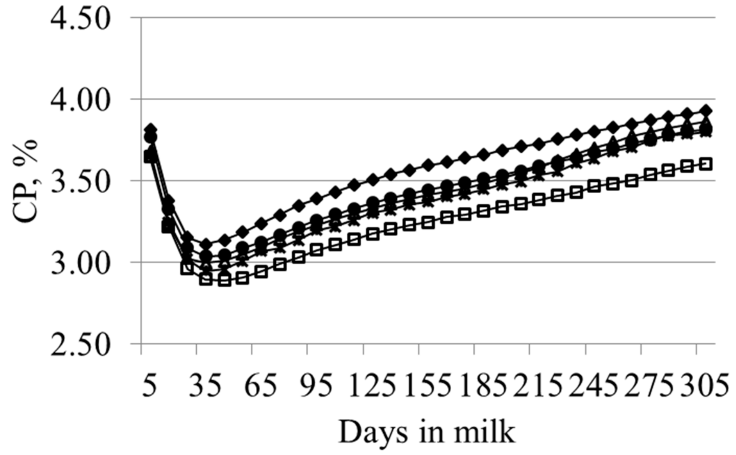

B

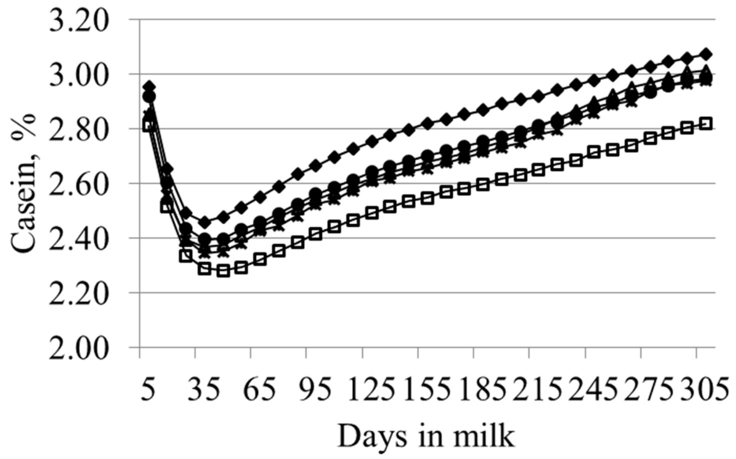

C

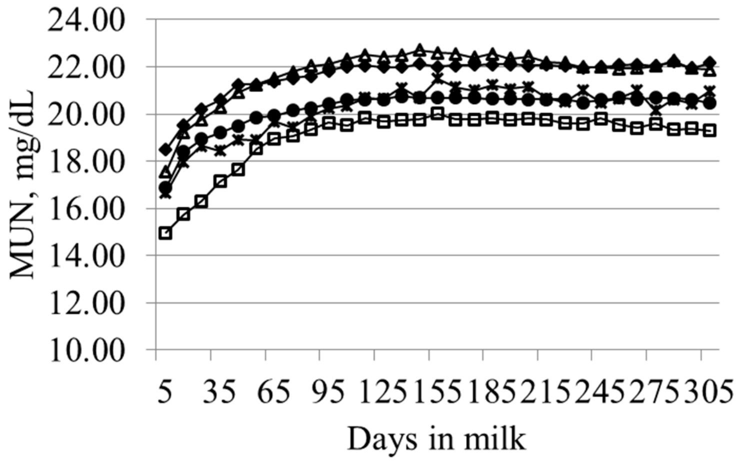

Figure S3. Least squares means of (**A**) crude protein (CP), (**B**) casein and (**C**) milk urea nitrogen (MUN) across month of sampling for Brown Swiss (-♦-), Holstein-Friesian (-□-), Alpine Grey (-△-), Simmental (-●-) and Pinzgauer (-×-) cows.

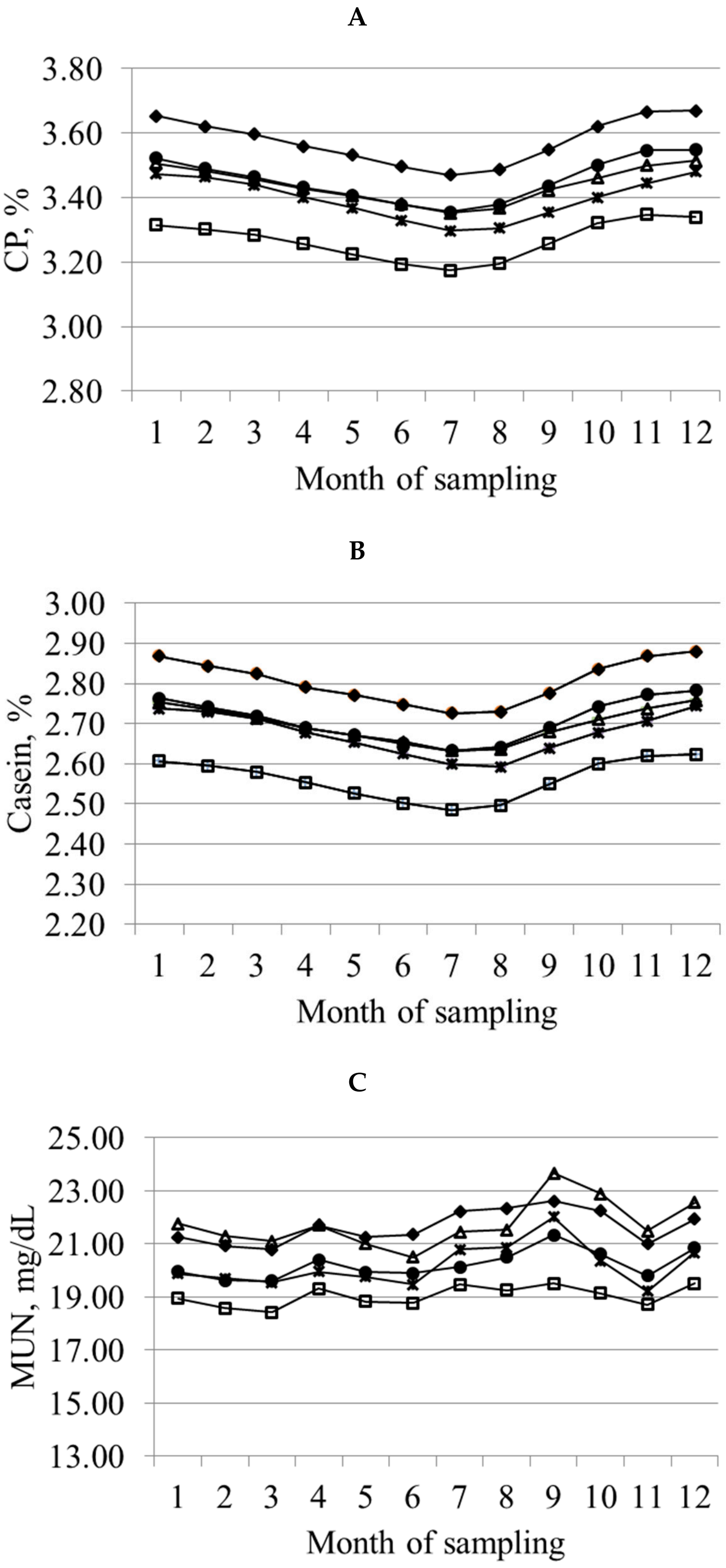

Supplement: Supplementary file 1 [file animals-09-00176-s001.pdf]
